# Integrity and resilience in the face of multiple crises: commentary from Puerto Rico

**DOI:** 10.1080/20961790.2021.1972904

**Published:** 2021-10-11

**Authors:** Richard J. Noel Jr, Jose A. Torres-Ruiz

Since 2010, the population of Puerto Rico has declined by 11.8% and is currently 3.3 million people [1]. This worrisome trend is a result of a plethora of island-wide challenges spanning financial difficulties, natural disasters, and epidemics. Following over a decade of economic hardship and debt, incapacitating hurricanes, Irma and Maria, destroyed large swaths of the island in 2017. While still recovering from that disaster, a magnitude 6.4 earthquake centered about 5 miles off the southwestern coast on January 7, 2020, destroying buildings and roads in southwestern Puerto Rico. This event, and subsequent aftershocks that continue until today, further aggravate an already stressed population. In the mix, viral epidemics have brought further economic, social, and health-related suffering. Puerto Rico was the centre of the 2016–2017 Zika virus infections, representing over 85% of the US infections in 2016 [2].

As with the rest of the world, the COVID-19 pandemic has demanded resilience from the population at large and specifically among the healthcare and research professionals working on their behalf. These long-standing hardships and more recent disasters have provided an opportunity for Ponce Health Sciences University (PHSU) to emerge as a leader in the region. Not only has this strengthened our sense of empathy for those facing similar challenges on a constant basis but it has helped us glean insights that may translate regionally or globally. For others facing such challenges, which are often compounded by corruption, poverty, struggling democracies, and other problems, our experience has offered some simple lessons in resiliency that we would like to share here.

As the largest of the four medical schools in Puerto Rico and the leading academic institution for biomedical and clinical research in the southwestern region of Puerto Rico, PHSU faces the daunting task of addressing health and social challenges for a large segment of the island’s population and across a wide geographical region. Fortunately, we have marshalled institutional and extramural resources to address the integrated healthcare and research needs of our people. National Institutes of Health (NIH) funding has been a major catalyst to enhancing institutional capacity at Ponce to conduct high-quality research designed to prevent and eliminate health disparities among our diverse population. The past and current work of the Research Centres in Minority Institutions (RCMI)-Specialized Centre for Health Disparities at PHSU, funded by National Institute on Minority Health and Health Disparities (NIMHD) (grant MD007579-36), has provided the platform to allow our scientists and medical professionals to serve our communities in meaningful ways.

The RCMI programme began at PHSU in 1985 as an infrastructural support programme from the US National Centre for Research Resources to develop biomedical research capacity and foster the development of a graduate programme offering a PhD in the Biomedical Sciences. Over the years, the programme has expanded in impact, providing essential resources to develop technical capacity, intellectual expertise, and critical infrastructure. In its current iteration as the RCMI-Specialized Centre for Health Disparities at PHSU, the centre supports three large research projects, a community engagement core, a large research resources component and infrastructure to support the development of junior investigators. The projects address health disparities in three domains: a basic science project that probes the impact of early life trauma and cocaine use on post-traumatic stress disorder, a clinical project to study postnatal consequences of perinatal Zika virus exposure, and a behavioural project studying the link between biopsychosocial stress and inflammation in breast cancer progression in Puerto Rican women. The community engagement component links a training programme for community researchers with PHSU scientists to facilitate community-based participation in development of research projects that address health disparities as identified by community leaders. A core to provide research resources, particularly instrumentation, as well as an investigator development core (called “START” for Strategic Academic Research Training) also form part of the Centre to support and complement the projects and cores. Finally, the entire Centre is coordinated through an administrative arm led by two principal investigators (PIs) and a programme coordinator.

As PIs, with critical support from our staff and each of the core and project components, we have sought to lead responsibly through these times of natural disasters and epidemics. We believe that showing consideration for our students, staff, faculty and research participants and being transparent about our decisions has helped to foster resilience and solidarity. In turn, this has meant we could offer almost uninterrupted service to our community while continuing our research and educational efforts. With this commentary, we seek to offer practical guidance for the global community of investigators currently facing difficulty, especially those whose infrastructure is compromised by compounding factors such as financial strain, political strife, or natural disasters, along with the pandemic. Forensic scientists working in disaster and crisis settings likely face similar circumstances every day in their ongoing work. It is our hope that our comments resonate with their experiences and that we may offer insights for those embarking on careers in the field.

## Natural disasters and epidemics on top of an enduring financial crisis

Puerto Rico, as a US territory, has not recovered from the great recession that impacted the nation in the late 2000s. Thus, in 2017 amid an already sub-par infrastructure, hurricane Maria caused extensive damage and devastation to both public and private property, transportation and utilities that left thousands of homeless and without basic resources across the island and billions of dollars in financial losses. Ponce is the largest city in Puerto Rico outside the San Juan/Metro region and is located on the southern coast of the island. The city and surrounding region suffered substantial additional infrastructure and financial losses due to the 2020 earthquakes—leading additional thousands of people to seek shelters or even sleep outside in tents for several months due to damaged homes and enduring fear caused by the aftershocks.

In addition to marshalling resources to help the regional communities, we have been able to quickly adapt and provide all possible institutional support for our researchers’ work and their students learning to advance and even thrive. Since the Zika epidemic in Puerto Rico and the devastation of hurricane Maria, the RCMI Programme at PHSU has doubled in size and supported the recruiting of five new junior research faculties. In parallel, PHSU has grown academically—increasing the size of its medical programme by 50% and expanding our other health-related schools and programmes. Throughout the difficult times, students across the School of Medicine, the School of Behavioral and Brain Sciences and the Public Health Programme have worked alongside our research faculty to provide urgent care and support to many affected and vulnerable communities—all while meeting their academic load and graduating on time.

## Diversity drives resilience in the face of a crisis

A strength of PHSU that is key to our ability to step in during a crisis lies in diversity and inclusion. With inclusion of those from diverse ethnic backgrounds and a healthy gender balance among our investigators and staff, we have sought to create an environment that contributes to the integrity and value of our research. This demographic diversity in turn supports our discipline diversity in research. For example, the research projects and cores in our Specialized Centre for Health Disparities blend expertise of scientists from public health, clinical, psychological and basic science backgrounds—all respectfully combining their strengths to maintain productivity through crisis to disaster and now in the pandemic. Thoughtful collaboration has become inherent to each of our translational projects, reflecting multidisciplinary approaches to priority questions and enabling us to learn and benefit from those in other settings. The combined knowledge and collegial spirit of these experts has allowed us to maintain resolve and persevere in our research, student training, and service missions as individuals and as an institution.

## Offering tailored support for junior faculty

Even given our inherently supportive institutional culture, these crises have required more from leadership to identify individual investigator needs and provide tailored solutions to allow our research teams to thrive. A good example derives from the burden the pandemic has placed on caregivers for children, elderly, immunocompromised individuals, and others, disproportionately affecting women and early career scientists. As leaders, we have had to proactively examine the need for additional support for specific faculty and staff to help them maintain their academic productivity, professional development, and overall well-being during times of increased stress and anxiety. Compounding external stressors, much of their anxiety has resulted from competing demands and a shifting balance between personal and professional roles.

In this effort, support for early career researchers has been demonstrated through the START programme as well as other initiatives, including those directly related to projects and more broadly as part of mentoring of trainees. We had to offer flexibility for our junior faculty in the START programme as far as meeting professional development requirements and progress markers. Of course, in 2020 the COVID-19 pandemic necessitated a switch to remote participation in our required monthly training workshops. While this worked well for some of the START faculties, many with school-age children had to balance time and internet bandwidth to accommodate their remote schooling. For such faculty, we recorded seminars so these START participants could benefit from the activity while balancing domestic responsibilities and scarcity of resources due to living in an area without sufficient broadband access. This was a simple and obvious solution implemented in many parts of the world. What is important is that leaders must look at such issues with compassion, flexibility, and an eye toward individual needs.

## Upholding our ethical responsibility to research participants and being transparent with our funders

The combined disasters and pandemic caused unprecedented complications for our studies that rely on research participant retention or new recruitment. Notably, RCMI researchers on the Zika virus cohort have largely overcome barriers to retention, maintaining over 91% of study participants across the past 3 years despite earthquakes and the ongoing pandemic. The researchers identified a creative strategy to maintain contact with study participants and implemented flexibility in scheduling annual physical and neurological exam appointments. Telemedicine strategies were helpful in assuring full participant compliance and linking participants with study staff. Diversity of expertise proved critical on the project leadership team, which consists of a pediatrician, a clinical psychologist and a microbiologist. We also found the resources to bridge gaps and maintain employment of key personnel to address the inevitable delays caused by loss of infrastructure due to earthquakes and need for reduced indoor capacity in the clinic due to COVID-19.

In contrast, the behavioural project studying biopsychosocial stress in cancer met severe hardship in participant recruitment, particularly because of the pandemic. This project involves interviews and revealing sensitive details that were not conducive to a remote setting. In coordination with our institutional review board, we set up a safe protocol and outfitted a designated space with protective shielding and other public health precautions to allow partici­pant recruitment for this and other projects requiring interaction with participants. However, the additional concerns of cancer patients, many of whom are immunocompromised, caused further delays in enrollment until vaccines became available in Puerto Rico in early 2021. PHSU which had built a strong reputation for leadership and community service throughout the Zika epidemic, hurricane Maria and the earthquakes that affected our region, became a leading site for COVID-19 testing and then a vaccine centre. We were able to capitalize on PHSU’s good reputation in the community as a trusted site to expedite vaccine availability and so helped to foster trust among potential study partici­pants in this cancer-related project. This solution, albeit slower to produce results, was one way we helped our behavioural project to resume recruitment more quickly by marshalling various institutional resources. Fundamental to our approach has been honest, open communication with our funders and external advisors. Through our transparency, we have been able to solicit additional support and suggestions for overcoming the obstacles we have faced.

## Paying attention to mental health in the face of consecutive stressful circumstances

As mentioned, PHSU built upon its reputation by taking action to address a multitude of consecutive crises through humility and compassion. The fact that through this period of sustained stress, we have grown as an institution, is a testament to the resi­lience of our faculty and staff and the integrity of our work in education, research and service. A criti­cal part of the longevity of our success in the face of a string of hardships has been our focus on the individual staff and faculty in our institution. Above we highlighted steps taken to address needs of our junior faculty through the START programme.

We realized we needed to extend our attention to also include more senior research faculty, along with students and staff. Realizing that we are all human and absorb stress and anxiety in different ways, we sought to offer respect and flexibility. This allowed members of the PHSU community to maintain ba­lance while dealing simultaneously with hardships at work and in their personal lives and yet still accomplish our work at a high standard and on time. Equally essential has been our collegial and collabo­rative institutional culture. We relied on the diversity of expertise among our professionals; for example, those in the School of Brain and Behavioral Sciences with expertise in crisis management convened forums to offer each other moral support. We also arranged for confidential access to trained psychological support for mental health issues among investigators, students, and staff to deal with the trauma and pressure inflicted by an epidemic, two major natural disasters and the pandemic. It has been an honour to witness the fact that unity and solidarity in such times have helped nurture our students, allowed us to retain faculty and staff, and made it possible to continue to secure extramural funding for our work.

## *Final point*s

We are a relatively small institution. A core element of our vision is to build upon our strengths by leading in diversity in education and research efforts focused on solving health disparities. Repeated disasters, whether financial, physical or infectious, have certainly brought crisis. We believe that during such crisis, emphasizing resilience and integrity has enabled us to remain humble while continue to serve our local and regional community in important ways. Our long-standing institutional culture that cherishes diversity and recognizes and rewards collegiality and collaboration, seeing each person as a cherished human being, have been essential to our ability to survive and even thrive. During times of crisis, decisive action by leadership is required. This usually means pivoting quickly and finding new ways to meet programme goals. We believe this also means listening to individual needs, offering customized solutions, and treating our people fairly while maintaining high expectations of integrity and accomplishment. There must also be a willingness to communicate openly with each other and with sponsors, research participants, and collaborators who rely upon our work.

Like many others in strained settings around the world, we have had to draw upon our individual and collective resolve. Our commitment to caring for our people while maintaining the rigour of our studies has enabled us to maintain the trustworthiness of our research. We hope our comments will be useful for other researchers facing similar plights, particularly when crises are compounded by each other and devasting to entire communities.


Richard J. Noel Jr
*Department of Basic Sciences, School of Medicine, Ponce Health Sciences University, Ponce, PR, USA*

Jose A. Torres-Ruiz
*Office of the Chancellor, Ponce Health Sciences University, Ponce, PR, USA*


